# The Performance of Diagnostic Tests for Severe Acute Respiratory Syndrome Coronavirus 2 (SARS-CoV-2) in the South African Population: A Scoping Review

**DOI:** 10.3390/tropicalmed8120514

**Published:** 2023-12-01

**Authors:** Natasha Samsunder, Nikita Devnarain, Aida Sivro, Ayesha B. M. Kharsany

**Affiliations:** 1 Centre for the AIDS Programme of Research in South Africa (CAPRISA), Durban 4013, South Africa; natasha.samsunder@caprisa.org (N.S.); nikita.devnarain@caprisa.org (N.D.); aida.sivro@caprisa.org (A.S.); 2 School of Health Science, University of KwaZulu-Natal, Durban 4013, South Africa; 3 Department of Medical Microbiology, University of KwaZulu-Natal, Durban 4013, South Africa; 4 JC Wilt Infectious Disease Research Centre, National Microbiology laboratory, Public Health Agency of Canada, Winnipeg, MB R3E 3L5, Canada; 5 Department of Medical Microbiology and Infectious Diseases, University of Manitoba, Winnipeg, MB R3T 2N2, Canada

**Keywords:** SARS-CoV-2, SARS-CoV-2 gene/s, diagnostic testing, RT-PCR, antigen, antibody, scoping review

## Abstract

To determine the performance and reliability of diagnostic tests for the identification of SARS-CoV-2 infection in South Africa, we conducted a scoping review to identify published studies undertaken in the English language from March 2020 to August 2022 that evaluated the performance of antigen- and antibody-based diagnostic tests for SARS-CoV-2 in South Africa. We identified 17 relevant peer-reviewed articles; six reported on SARS-CoV-2 gene and/or antigen detection whilst 11 reported on antibody detection. Of the SARS-CoV-2 gene and/or antigen-based tests, sensitivity ranged from 40% to 100%, whilst for the antibody-based tests, sensitivity ranged from 13% to 100%. All tests evaluated were highly dependent on the stage of infection and the timing of sample collection. This scoping review demonstrated that no single SARS-CoV-2 gene and/or antigen- or antibody-based assay was sufficiently sensitive and specific simultaneously. The sensitivity of the tests was highly dependent on the timing of sample collection with respect to SARS-CoV-2 infection. In the case of SARS-CoV-2 gene and/or antigen detection, the earlier the collection of samples, the greater the sensitivity, while antibody detection tests showed better sensitivity using samples from later stages of infection.

## 1. Introduction

The unprecedented spread of severe acute respiratory syndrome coronavirus 2 (SARS-CoV-2) resulted in the urgent need for rapid and reliable diagnostic tests. Accurately diagnosing individuals with infection was paramount to limit the transmission of the virus and to reduce morbidity and mortality. Whilst individuals exposed to SARS-CoV-2 appear to be equally at risk of acquiring infection, the severity of the resulting clinical disease differs markedly by age with a case fatality rate of <1% for people <60 years and sequentially increasing to 14.8% among those 80 years or older [1].

Based on the first available SARS-CoV-2 viral sequences, the World Health Organization (WHO) issued guidance on polymerase chain reaction (PCR)-based assays to be performed from upper respiratory tract specimens as the “gold standard” for the detection of SARS-CoV-2 infection [2]. Africa, and in particular, South Africa, relied on the existing PCR-based platforms that had been established for human immunodeficiency virus (HIV) and tuberculosis (TB), enabling the rapid introduction and scale-up of testing for SARS-CoV-2 infections [3]. Notwithstanding South Africa’s diagnostic capabilities to undertake testing, commercially available diagnostic tests and consumables including competitive first-world pricing and prioritisation to specific institutions were a major challenge in accessing and scaling up testing services to address the rapidly growing needs of the country to determine the extent of current and past infection. 

The evolution of the rRT-PCR is based on primers and probes (nCoV_IP2 and nCoV_IP4) that were designed to target the genes that encode for the nucleocapsid (N), envelope (E), spike (S), and RdRp proteins [4]. Rapid diagnostic tests (RDT) were designed and to be point-of-care (POC) tests for the detection of SARS-CoV-2 gene/s and/or antigens that are simpler to perform and have a shorter turnaround time. There is minimal evolution of the N gene and therefore most POC tests target the nucleocapsid. Once the virus has entered the host cell, it releases its genomic mRNA material in the cytoplasmic compartment and the translation of ORF-1a and ORF-1b begins [5]. This is followed by viral RNA expression and the replication of genomic RNA to produce full-length copies that are incorporated into newly produced viral particles [6]. Individuals with SARS-CoV-2 infection elicit an innate immune response within hours of viral exposure, followed by the development of Immunoglobulin M (IgM) and Immunoglobulin G (IgG) antibodies at around 7 to 14 days [2,7]. Thus, the detection of SARS-CoV-2 gene/s and/or antigen and antibody responses helps in understanding the infectiousness, transmission dynamics, and natural history of the disease. 

Viral shedding has been found to occur in oropharyngeal and nasal or sputum, tracheal aspirates, bronchoalveolar lavage and saliva [8], faeces, urine [9], and semen samples [10]. These findings highlight the need for alternative sampling approaches to improve diagnostic performance and to understand the magnitude and/or duration of viral shedding that could correlate with disease severity and viral dynamics to influence infection and transmission outcomes. The rapidly evolving SARS-CoV-2 pandemic with the emergence of new SARS-CoV-2 variants and subvariants has led to complex diagnostic testing challenges, especially in settings with limited access to diagnostic tests. 

This scoping review evaluated the laboratory performance of SARS-CoV-2 diagnostic tests in South Africa to identify knowledge gaps and enhance the accuracy of these tests. 

## 2. Materials and Methods

This scoping review followed the PRISMA-ScR (Preferred Reporting Items for Systematic reviews and Meta-Analyses extension for Scoping Reviews) checklist [11] and followed the framework of Levac et al. [12]. 

### 2.1. Eligibility Criteria

Included in this review were articles reporting original studies undertaken in South Africa between March 2020 to August 2022, peer-reviewed and published in the English language. Articles examining other diseases or behaviours related to mitigating SARS-CoV-2 transmission, with incomplete data, or which were either opinion pieces, reviews, or guidelines and not undertaken in South Africa were excluded. 

### 2.2. Information Sources

Two independent reviewers (NS and ND) designed a search strategy and systematically searched bibliographic databases—the PubMed, Web of Science, and Scopus electronic databases—for published articles. Manual searches were conducted by reviewing the references of published articles. The final search results were exported into Endnote^TM^20 (Thomson Reuters, New York, NY, USA) software, a reference management tool for citations.

### 2.3. Search Strategy

The search strategy terms used individually and/or in combination included “severe acute respiratory syndrome coronavirus 2”, “SARS-CoV-2”, “testing for SARS-CoV-2 in South Africa”, “SARS-CoV-2 antibody”, “testing for COVID-19”, real-time reverse transcriptase–polymerase chain reaction for SARS-CoV-2, rRT-PCR for SARS-CoV-2, “SARS-CoV-2 PCR”, “SARS-CoV-2 GeneXpert”. 

### 2.4. Data Charting and Extraction

Each reviewer (NS and ND) screened the article titles and abstracts independently, excluded duplicate articles, merged the results of the review, and resolved discrepancies. The final set of full articles was reassessed for the pre-set inclusion criteria. The reviewers prepared the data charts, standardised the data abstraction process, and independently charted the data for discussion and the final selection. Disagreements were resolved through discussion for the final selection of articles.

### 2.5. Quality Assessment

Eligible articles were evaluated using the quality appraisal tool [13] and scored to assess clarity, the sampling and data collection strategy, the sample representative of the target population, measurements, the risk of non-response, and statistical analysis to address the research question. NS assessed the quality of the studies as being of low quality (score ≤ 50%), average quality (51% to 75%), and high quality (76% to 100%). No articles were excluded based on quality. 

## 3. Results

### 3.1. Study Selection

**Figure 1:**
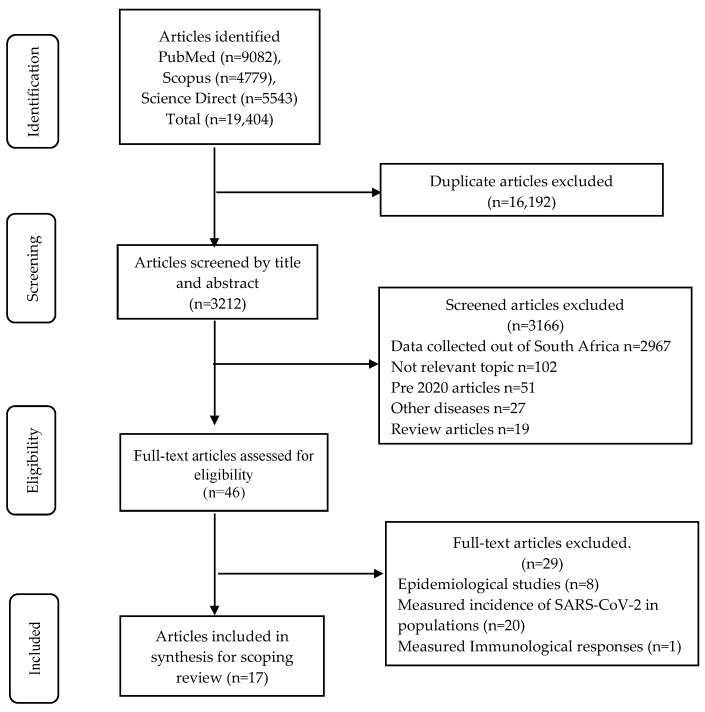
Search strategy and selection of articles for data extraction, analysis, and reporting for a scoping review according to PRISMA-ScR guidelines.

Figure 1 shows the identification, screening, eligibility, and inclusion of the studies for the scoping review [14,15,16,17,18,19,20,21,22,23,24,25,26,27,28,29,30]. The quality assessment of the 17 articles resulted in four articles scoring 71% and the rest scoring 86% and above. Table 1 provides an overview of the testing kits or methodology evaluated. Seven of the 17 studies included populations from Gauteng [17,19,21,22,24,29,30], four from the Western Cape [15,16,26,28], one from Limpopo [14], one from Eastern Cape [18], and one from the Free State [23]. Of the remaining three studies, two studies included samples from more than one province [20,27] and one study undertook testing on stored samples [25]. All studies were cross-sectional in design and tested samples retrospectively or prospectively. The majority of the studies were undertaken with well-characterised samples from patients experiencing COVID-19 at different stages of the disease, thus allowing for sensitivity assessment of the tests over specific time periods. Several studies describing antibody kit evaluations included archived pre-COVID-19 samples to assess specificity. Samples were collected starting from the origin of the D614G strain, followed by the Beta, Delta, and Omicron variants. Table 2 provides the performance characteristics of the SARS-CoV-2 diagnostic tests, which are either commercially available or are in-house assays, the manufacturer, country of manufacture, analytes measured, sample type, sample volume required per test, time taken to perform the test to obtain the result, and the complexity of the testing procedure. Not all studies included the volume of sample required, though they indicated the sample type used. Table 3 shows the analytical assessment and testing parameters of SARS-CoV-2 gene(s) and antigen- and antibody-based diagnostic tests. 

### 3.2. SARS-CoV-2 Gene and Antigen-Based Diagnostic Tests

Studies that evaluated diagnostic tests for the detection of SARS-CoV-2 genes and antigens included RT-PCR variant genotyping [14], followed by the Allplex^TM^ SARS-CoV-2 Variants II multiplex real-time PCR genotyping assay by Seegene (Seoul, South Korea). The testing was based on circulating Beta and Delta variants prior to the emergence of the Omicron variant and utilised specific primers and probes for each variant. The results were available in two hours as opposed to the time-consuming next-generation sequencing. This assay delineated the Beta and Delta variants and had the ability to determine the rapid rate at which the Delta displaced the Beta variant in the study setting of Limpopo, and thus the capability of the assay to rapidly monitor circulating variants [14]. The reproducibility of the assay was identical across operators with near identical cycle threshold (Ct) values, whilst the overall average Pearson correlation for linearity between the SARS-CoV-2 median Ct and variant typing Ct values for the samples analysed was 0.976 (standard deviation (SD) ±0.019) with 96.4% concordance for repeatability. However, testing was restricted to known circulating variants. 

To improve the turnaround time and to be less reliant on reagents, equipment, and staff, Marais et al. [15] from the Western Cape applied a revised workflow using rapid sample preparation (RSP) with a key modification that included sample centrifugation and heating prior to RT-PCR for either the Abbott RealTime SARS-CoV-2 assay or the Allplex^TM^ 2019-nCoV assay platforms. This modification showed a 97.37% (95% confidence interval (CI):92.55–99.28) positive per cent agreement (PPA) and a 97.30% (95% CI:90.67–99.52) negative per cent agreement (NPA) compared to nucleic acid purification-based testing. In confirmed Delta variant infections, the PPA of RT-PCR on saliva was 73% (95% CI:53.0–84.0). 

In Omicron variant infections, saliva performed as well as or better than mid-turbinate samples up to day 5, with an overall PPA of saliva swabs of 96% and mid-turbinate samples of 93%, demonstrating the altered kinetics in viral shedding [16].

As the demand for diagnostic testing overwhelmed the capacity to deliver, Omar et al. assessed the utility of a mobile laboratory staffed with non-laboratory healthcare personnel to undertake PCR testing [17]. Using the 2400 SARS-CoV-2 Smartchecker PCR kit (Genesystem, Daejeon, South Korea) targeting the *N* and *RdRp* genes and processed using the thermocycler (Genechecker; Genesystem, Daejeon, South Korea) showed a median turnaround time of 152 min (interquartile range 123–184) with sensitivity and specificity of 95% and 97% and positive and negative predictive values of 82.4% and 99.2%, respectively, when compared to a clinical diagnosis of COVID-19. 

With increasing demands on testing for SARS-CoV-2 infection, two studies [18,19], evaluated the field performance of the Abbott Panbio Antigen Rapid Test Device (Ag-RDT) (Abbott, San Diego, Carlsbad, CA, USA) against the available SARS-CoV-2 RT-PCR, which detects the Beta and Delta variants [18,19]. In the Eastern Cape province, the test had a sensitivity of 69.17% (95% CI:61.4–75.8) and specificity of 99.02% (95% CI:98.8–99.3) among symptomatic individuals [18], whilst among members of the public at three taxi ranks in Johannesburg, the test had a sensitivity of 40.0% (95% CI:30.3–50.3) and specificity of 98.5% (95% CI:96.9–99.4) with a positive predictive value of 85.1% (95% CI:71.7–93.8) and a negative predictive value of 88.5% (95% CI:85.5–91.1) [19]. The sensitivity of the test was dependent on the amount of viral RNA in clinical samples, as reflected by the PCR Ct value [19].

### 3.3. SARS-CoV-2 Antibody-Based Diagnostic Tests

Serological assays for the detection of IgG, IgM, or Immunoglobulin A (IgA) against SARS-CoV-2 infection provide important information for surveillance, antibody persistence, infection rate, and vaccine coverage. Serological assays, including enzyme-linked immunosorbent assays (ELISA) and rapid lateral flow assays, are available commercially; however, the high cost limits their accessibility in resource-limited countries. Although several assays have been developed, field evaluations have been limited. Testing was performed on serum samples, plasma, fingerstick, and dried blood spot (DBS) samples. The analytical assessments of antibody-based diagnostic tests for SARS-CoV-2 are shown in Table 3. Of the twelve studies, five (38.5%) compared serological outcomes to RT-PCR, whilst in seven (54%), a comparison was made to either in-house or commercially available serological tests.

Makatsa et al. (2021) [20] developed an in-house indirect ELISA using plant-derived recombinant viral proteins by means of the S1 and receptor-binding domain (RBD) portions of the spike protein from SARS-CoV-2, expressed in *Nicotiana benthamiana* [20]. This test measured antibody responses among SARS-CoV-2 PCR-positive patients. Samples taken at a median of 6 weeks from diagnosis from patients with mild and moderate COVID-19 disease showed that the in-house ELISA, when compared to the S1 IgG ELISA kit (EUROIMMUN), detected immunoglobulins; S1-specific IgG was detected in 66.2% and RBD-specific IgG in 62.3% of samples and were concordant with the EUROIMMUN assay. 

To optimise the diagnostic algorithm for SARS-CoV-2 infection, Gededzha et al. (2021) [21] evaluated the diagnostic performance of the EUROIMMUN Anti-SARS-CoV-2 ELISA for the semi-quantitative detection of IgA and IgG antibodies in serum and plasma samples targeting the recombinant spike (S1) domain of the SARS-CoV-2 spike protein as the antigen. The sensitivity of EUROIMMUN was higher for IgA (74.3%, 95% CI:69.6–78.6) than for IgG (64.1%, 95% CI:59.1–69.0), though specificity was lower for IgA (84.2%, 95% CI:77–89.2) than IgG (95.2%, 95% CI:90.8–98.4) and both sensitivity and specificity improved in symptomatic individuals [21]. 

The performance of the Abbott SARS-CoV2 Architect and Abbott SARS-CoV2 Alinity IgG when compared to RT-qPCR showed the sensitivity of the assays to be 69.5% (95% CI:64.7–74.1) and 64.8% (95% CI:59.4–69.9), respectively, whilst the specificity of the assays was 95% (95% CI:89.9–98) and 90.3% (95% CI:82.9–95.2), respectively. When the assays were compared to the in-house ELISA, the sensitivity for the Architect and Alinity assays was 94.7% (95% CI:88.8–98) and 92.5% (95% CI:85.8–96.7), respectively, whilst specificity was 88.1% (95% CI:79.2–94.1) and 91.7% (95% CI:83.6–96.6), respectively. The sensitivity for both assays was highest at 31–40 days post-presentation and lowest at time points of less than 7 days. These findings highlight the futility of testing for antibody responses during the acute and early stages; that is, within less than 14 days of infection [22]. 

Matefo et al. (2022) investigated two in-house ELISAs and an in-house immunofluorescent assay (IFA), developed using the SARS-CoV-2 S1 protein, for use in South African populations [23]. The tests were compared with Roche Elecsys^TM^ Anti-SARS-CoV-2 (Roche Diagnostics GmbH, Mannheim, Germany) and a commercial lateral flow assay, COVID-19 IgG/IgM Rapid Test cassette (Zhejiang Orient Gene Biotech Co., Ltd., Zhejiang, China). Based on IgG antibodies, specificity was 96% and 100% for ELISA and IFA, respectively, and sensitivity was shown to be 100% and 98.8% for ELISA and IFA, respectively, for samples collected one week after the onset of illness. Positive predictive values were 92.1% for ELISA and 91.0% for IFA. The in-house ELISA and IFA were positive for IgG antibodies, regardless of circulating variants, therefore demonstrating the potential of these tests for high throughput screening in resource-constrained environments [23]. 

The performance of the Roche Elecsys^TM^ chemiluminescent immunoassay (Rotkreuz, Switzerland) to detect antibodies to SARS-CoV-2 N as antigen was evaluated by Grove et al. [24]. Among patients from Johannesburg, serum samples from SARS-CoV-2 RT-PCR positive and negative individuals showed a sensitivity of 65.2% (95% CI:59.57–70.46) and specificity of 100% (95% CI:97.07–100). The sensitivity of the test improved to 72% among those with >14 days and to 88.6% in those 31–50 days post diagnosis. Nevertheless, using the in-house ELISA assay utilising the plant-based S1 and RBD as antigens [20], the overall PPA was 89.4% (95% CI:82.18–94.39) and NPA was 88.4% (95% CI: 80.53–93.83). However, among individuals at earlier time points post-infection and among asymptomatic individuals, the sensitivity was lower with the Roche Elecsys^TM^ chemiluminescent immunoassay and the in-house ELISA [24]. 

David et al. (2021) evaluated 30 lateral flow immunoassays using serum or plasma samples from patients with confirmed SARS-CoV-2 infection [25]. Of these, 26 assays did not meet the predefined operational acceptance criteria for kits to be approved for use in South Africa. Whilst the performance of the lateral flow tests was similar to the sensitivities and specificities reported in other studies, only four (13%) assays (Zheihang Orient Gene COVID-19 IgG/IgM, Genrui Novel Coronavirus (2019-nCoV) IgG/IgM, Biosynex COVID-19 BSS IgG/IgM, Boson Biotech 2019-nCoV IgG/IgM) were recommended for South Africa Health Products Regulatory Authority (SAHPRA) approval [25]. 

Among volunteers in Cape Town, 23.7% tested positive for IgG antibodies with the Abbott SARS-CoV-2 IgG assay. Of those who tested positive, 47.9% reported no symptoms of COVID-19 in the past 6 months. Seropositivity was significantly associated with living in informal housing, residing in a subdistrict with low income per household, and having a low-earning occupation. The specificity of the assay was 98.54% (95% CI:94.82–99.82) [26].

In the household survey undertaken in three communities across three provinces in South Africa, the burden of SARS-CoV-2 infections was measured using two ELISA kits: Wantai SARS-CoV-2 Ab ELISA (Beijing Wantai Biological Pharmacy Enterprise), measuring total antibodies (IgM, IgG and IgA) against the RBD in the spike protein, and Roche Elecsys^TM^ Anti-SARS-CoV-2 ELISA (Roche Diagnostics), measuring total antibodies to the N protein. There was 94.5% PPA with a Cohen κ statistic of 0.89. The Wantai assay, compared with the Roche Elecsys^TM^ assay, had a sensitivity of 91.0% and a specificity of 97.2% [27] 

To monitor antibody responses to SARS-CoV-2 following a vaccine rollout, Maritz et al. (2021) assessed a ligand binding-based serological assay for the semiquantitative detection of IgG, IgM, IgA, and neutralising antibodies (nAb) in serum [28]. The assay demonstrated high levels of diagnostic specificity and sensitivity (85–99% for all analytes). Serum IgG, IgM, IgA, and nAb correlated positively (R2 = 0.937, R2 = 0.839, R2 = 0.939 and R2 = 0.501, *p* < 0.001, respectively) with those measured in DBS samples. In vitro SARS-CoV-2 pseudotype neutralisation correlated positively with the solid phase nAb signals in convalescent donors (R2 = 0.458, *p* < 0.05), highlighting the potential use of the assay in efficacy studies, infection monitoring, and post-marketing surveillance following vaccine rollout [28].

To enable large-scale testing for SARS-CoV-2 antibodies, DBS samples were evaluated against plasma samples with a correlation of r = 0.935 and 0.965 for RBD and full-length S-protein of SARS-CoV-2 [29]. A Bland–Altman assessment showed agreement between IgG mean fluorescence intensity (MFI) values with 6.25% of observations for both RBD IgG and spike IgG, falling outside the 95% limit of agreement. Therefore, DBS samples are a useful medium for population screening and field studies in resource-constrained settings, as they are non-invasive and ideal for storage, transportation, and processing [29].

As the need for testing increases, especially for surveillance or during outbreak situations, rapid antibody testing assays are useful in such situations. Irwin et al. (2021) evaluated the sensitivity of five rapid antibody assays and explored factors influencing their sensitivity in detecting SARS-CoV-2-specific IgG and IgM antibodies [30]. In addition, finger-prick blood samples from participants within 2–6 weeks of PCR-confirmed COVID-19 diagnosis were included in the evaluation. Overall sensitivity for IgG and IgM antibodies was below 70% and ranged from 13% to 67% for IgG and markedly lower for IgM. Whilst rapid tests in resource-constrained settings are a promising tool in COVID-19 diagnosis, the sensitivity was reduced for those under 40 compared with those over 40 years of age. These findings show significant variability when used in real-world settings, limiting their application [30]. 

## 4. Discussion

The scoping review yielded 17 studies that assessed the performance of diagnostic tests in South Africa for the detection of SARS-CoV-2 infection. These studies were important, especially during the unprecedented spread of SARS-CoV-2 in diverse populations and in a high HIV- and TB-burden setting. Therefore, this scoping review provided an opportunity for an analytical assessment of rapidly emerging diagnostic tests, especially for a newly identified virus, complicated by the rapid evolution of novel variants and sub-variants. 

Whilst the international community made considerable progress to produce and distribute diagnostic tests, access to high-quality testing platforms was extremely limited in low- and middle-income countries. Additionally, the need for in-country regulatory approvals by SAHPRA prior to the utilisation of diagnostic tests contributed to substantial delays in the availability of such tests, diminishing the urgency of testing and perpetuating the risk of onward community transmission. 

Several important insights and themes emerged from this scoping review. All studies identified were deemed to be of high quality and were from diverse studies. These studies demonstrated that the timing of the collection of clinical samples with respect to symptom onset had a major impact on assay sensitivity. The sensitivity of the SARS-CoV-2 gene/s and antigen detection tests improved when samples were collected during the earlier stages of infection when the SARS-CoV-2 viral load was the highest. As expected, sensitivity declined when samples were collected during the advancing stages of infection. However, there was no consensus on the precise timing of sample collection, and therefore, SARS-CoV-2 negative rapid test results in suspected cases require further testing to confirm the results [14,15,16,29]. Importantly, the quality of the nasopharyngeal, oropharyngeal, and nasal swabs or tracheal aspirates collected is dependent on the skills and expertise of the medical personnel, directly impacting test performance. Furthermore, sampling for POC self-tests by untrained, non-medical staff would be an influencing factor for the sensitivity of the tests resulting in misleading results. 

For the SARS-CoV-2 gene/s and antigen testing evaluations, the Allplex^TM^ SARS-CoV-2 Variants II multiplex real-time PCR genotyping assay addressed the genotyping challenges, was simpler to perform, easier to interpret, and was less expensive than conventional genotyping. Improving the sample preparation had the added advantage of improving the turnaround time for test results with the capability of handling samples during peak testing periods. Furthermore, the assay provided evidence of recombination or mixed populations of variants identified through low *S* gene Ct values that failed to be assigned a variant, thus harbouring novel mutations and highlighting the need for confirmation with next-generation sequencing. 

The testing kits applied in the field nevertheless had a shortened turnaround time with improved sensitivities and specificities [17]. A limitation of these tests was the design of target molecules based on primers and probes to identify existing known variants, thus making them likely to miss new variants as and when they emerge. It is important that assays are designed to improve variant detection capacity and identify multiple variants, including novel variants, or include targets that are likely to be common to variants and subvariants of SARS-CoV-2. 

Our scoping review has some limitations. Whilst studies were of high quality, the sample sizes in several studies were relatively small. Furthermore, the utility of different sample types such as saliva demonstrated lower levels of sensitivity, reducing their use in settings that may require high throughput even though saliva sample collection might be easier. This review highlights the limited number of test evaluations that have been conducted in South Africa, with the majority of studies taking place in Gauteng and the Western Cape. With the diverse population, different environmental conditions, and other infectious diseases prevailing in various parts of South Africa, it is important that diagnostic tests be evaluated prior to implementation in local settings and specifically across all provinces. The variability in the studies included in the scoping review makes it difficult for direct comparisons, therefore the selection of laboratory tests should be based on the laboratory evaluation of the test kits as part of the initial evaluation to include panels of samples collected over time from more than one province. This should be followed by field evaluation of the tests, as real-life use may highlight certain challenges and nuances that may not be observed in controlled laboratory testing by trained laboratory staff. In addition, the evaluation of test kits should be expanded to two or more provinces, considering the epidemiology of the analytes being tested, especially in settings with low and high prevalence to provide robust performance data prior to roll-out of testing. Another limitation was the lack of peer-reviewed publications appearing as preprints that were not included in this review. 

## 5. Conclusions

Our review indicates that timely diagnosis of SARS-CoV-2 is critical to reduce transmission, morbidity, and mortality. Although diagnostic tests for SARS-CoV-2 varied considerably in sensitivity, the duration of infection, the timing of sample collection, and SARS-CoV-2 Variants of Concern all impacted the sensitivity of diagnostic tests. These findings therefore highlight the importance of improvements to existing diagnostic tests or the application of broad-based epitopes in the next generation of diagnostic tests to enhance sensitivity and specificity.

## Figures and Tables

**Table 1:** Overview of articles on diagnostic testing for SARS-CoV-2 in South Africa.

Study (Author, Ref)	Province	Study Design	Date Range	Clinical Disease Stage	SARS-CoV-2 Viral Variants
**Genotyping**					
Umunnakwe et al., 2022 [14]	Limpopo	Cross-sectional random sampling of samples from biorepository	April–Oct 2021	Not reported	Beta, Delta
**RT-PCR**					
Marais et al., 2020 [15]	Cape Town, Western Cape	Cross-sectional retrospective testing of preselected samples	Not reported	Not reported	Not reported
Marais et al., 2022 [16]	Cape town, Western Cape	Prospective cross-sectional	20 August–19 November 2021 19 November 2021– 7 February 2022	Ambulatory outpatients	Delta, Omicron
Omar et al., 2021 [17]	Johannesburg, Gauteng	Retrospective descriptive cross-sectional	20 May–8 August 2020	Symptomatic Asymptomatic	D614G
**Rapid antigen testing**					
Akingba et al., 2021 [18]	Nelson Mandela Bay, Eastern Cape	Prospective cross-sectional evaluation	17–20 November 2020	Symptomatic	Beta
Majam et al., 2022 [19]	Johannesburg, Gauteng	Prospective evaluation	June–September 2021	Random sampling	Delta
**Serological testing**					
Makatsa et al., 2021 [20]	Gauteng and Western Cape	Prospective evaluation	10 April–26 May 2020	Symptomatic Asymptomatic	D614G
Gededzha et al., 2021 [21]	Braamfontein, Gauteng	Retrospective cross-sectional evaluation of volunteer samples	Not reported	87% symptomatic, 13% asymptomatic	Not reported
Jugwanth et al., 2022 [22]	Braamfontein, Gauteng	Cross-sectional sample of volunteers	Not reported	87% symptomatic, 13% asymptomatic	Not reported
Matefo et al., 2022 [23]	Bloemfontein, Free State	Retrospective cross-sectional of patient samples	March–October 2020		D614G
Grove et al., 2021 [24]	Johannesburg, Gauteng	Prospective analytical evaluation	May–August 2020	Not reported	D614G
David et al., 2021 [25]	Not reported	Retrospective cross-sectional testing on previously confirmed samples	Not reported	Not reported	Not reported
Shaw et al., 2021 [26]	Cape Town, Western Cape	Cross-sectional volunteers	17 August–4 September 2020	Volunteers	D614G
Wolter et al., 2022 [27]	Mitchell’s Plain Western Cape, Pietermaritzburg KwaZulu-Natal, Klerksdorp Northwest	Prospective cross-sectional household seroprevalence survey in 3 communities	March and April 2021	Not reported	Beta
Maritz et al., 2021 [28]	Stellenbosch, Western Cape	Retrospective cross-sectional of volunteer samples	Not reported	Asymptomatic	Not reported
Kwatra et al., 2022 [29]	Soweto, Gauteng	Retrospective sampling of participants testing positive	April–December 2020	Hospitalised Symptomatic	D614G, Beta
Irwin et al., 2021 [30]	Johannesburg, Gauteng	Cross-sectional of randomly selected in- and out-patients	Not reported	Symptomatic Asymptomatic	Not reported

**Table 2:** Performance characteristics of the diagnostic tests for SARS-CoV-2.

Study (Author, Ref)	Product Name	Manufacturer /Country	Analyte Measured	Sample	Sample Volume	Read Time	Complexity
**Genotyping**							
Umunnakwe et al., 2022 [14]	Allplex^TM^ SARS-CoV-2 Variants II multiplex real-time PCR genotyping assay	Seegene/South Korea	Specific primers and probes for Beta and Delta variants	Nasopharyngeal swab	Not applicable	~2 h	Slightly complex
**RT-PCR**							
Marais et al., 2020 [15]	Rapid sample preparation (RSP) Abbott RealTime SARS-CoV-2 Assay or Allplex^TM^ 2019-nCoV assay	Abbott Laboratories and Seegene	E, N, RdRp genes	Nasopharyngeal and oropharyngeal swabs	Not applicable	Not reported	Slightly complex
Marais et al., 2022 [16]	RT-PCR on saliva and mid-turbinate sample vs. respiratory swab	Abbott Laboratories/USA and Seegene/South Korea	E, N, RdRp genes	Saliva, mid-turbinate swab	Not applicable	8–12 h	Medium complex
	FlowFlex SARS-CoV-2 N protein lateral flow assay	ACON Laboratories Inc./USA	Nucleocapsid protein	Saliva, mid-turbinate swab	Not applicable	15 min	Technically simple
Omar et al., 2021 [17]	Thermocycler (Genechecker; and a 2400 SARS-CoV-2 Smartchecker PCR kit	Genesystem/South Korea	N, RdRp gene	Nasopharyngeal, oropharyngeal, nasal swabs, tracheal aspirates	Not applicable	45 min	Slightly complex
**Rapid antigen testing**							
Akingba et al., 2021 [18]	PanBio COVID-19 antigen test	Abbott Rapid Diagnostics/USA	Nucleocapsid protein	Nasopharyngeal swab	Not applicable	15 min	Technically simple
Majam et al., 2022 [19]	PanBio COVID-19 antigen test Device	Abbott Rapid Diagnostics/USA	Nucleocapsid protein	Nasopharyngeal swab	Not applicable	15 min	Technically simple
**Serological testing**							
Makatsa et al., 2021 [20]	Indirect in-house ELISA using recombinant plant-derived viral proteins	Cape BioPharms/South Africa	IgG	Serum	Not reported	Not reported	Slightly complex
Gededzha et al., 2021 [21]	EUROIMMUN Anti-SARS-CoV-2 IgA and IgG	EUROIMMUN Medizinische Labor diagnostika AG/Germany	IgG, IgA	Serum, plasma	Not reported	Not reported	Slightly complex
Jugwanth et al., 2022 [22]	Abbott SARS-CoV-2 IgG Architect	Abbott Diagnostics/USA	IgG	Serum, plasma	Not reported	Not reported	Slightly complex
	Abbott SARS-CoV-2 Alinity	Abbott Diagnostics/USA	IgG	Serum, plasma	Not reported	Not reported	Slightly complex
Matefo et al., 2022 [23]	Laboratory-developed ELISA and IFA	Not applicable	IgG	Serum	Not reported	Not reported	Complex
Grove et al., 2021 [24]	Roche Elecsys^TM^ chemiluminescent immunoassay	Roche Diagnostics/Switzerland	IgM, IgG	Serum, plasma	Not reported	Not reported	Slightly complex
David et al., 2021 [25]	Zheihang OrientGene COVID-19 IgG/IgM	Orient Gene Biotech/China	IgM, IgG	Venous blood	5 ul	10 min	Technically simple
	Genrui Novel Coronavirus (2019-nCoV) IgG/IgM	Genrui Biotech Inc/China	IgM, IgG	Venous blood	10 ul	10 min	Technically simple
	Boson Biotech 2019-nCoV IgG/IgM	Xiamen Boson Biotech/China	IgM, IgG	Venous blood	2 ul	10 min	Technically simple
	Biosynex COVID-19 BSS	Biosynex Swiss SA/Switzerland	IgM, IgG	Venous blood	10 ul	10 min	Technically simple
Shaw et al., 2021 [26]	Abbott SARS-CoV-2-2 IgG assay	Abbott Laboratories/South Africa	IgM, IgG	Whole blood	Not applicable	30 min	Slightly complex
Wolter et al., 2022 [27]	Wantai SARS-CoV-2 Ab ELISA	Wantai Biological Pharmacy Enterprise/China	IgM, IgG, IgA	Serum, plasma	Not reported	Not reported	Slightly complex
Maritz et al., 2021 [28]	Semi-quantitative detection for IgG, IgM, IgA and Nab in serum	Not applicable	IgM, IgA	Serum	Not reported	Not reported	Complex
Kwatra et al., 2022 [29]	DBS serology vs. plasma serology	Not applicable	RdRp gene S-protein	Dried blood spot, plasma	Not reported	Not reported	Slightly complex
Irwin et al., 2021 [30]	2019-nCoV-IgG/IgM Rapid Test	Dynamiker Biotechnology Company Ltd./China	IgM, IgG	Fingertip whole blood	10–20 ul	15–20 min	Technically simple
	2019-nCoV IgG/IgM Rapid Test Cassette	AllTest Biotech Company Ltd./China	IgM, IgG	Fingertip whole blood	10–20 ul	15–20 min	Technically simple
	2019-nCoV Ab Test (Colloidal Gold)	Innovita Biotechnology Company Ltd./China	IgM, IgG	Fingertip whole blood	10–20 ul	15–20 min	Technically simple
	Medical Diagnostech COVID-19 IgG/IgM Rapid Test	Altis Biologics (Pty) Ltd./South Africa	IgM, IgG	Fingertip whole blood	10–20 ul	15–20 min	Technically simple
	Cellex qSARS-CoV-2 IgG/IgM Cassette Rapid Test	Cellex/China	IgM, IgG	Fingertip whole blood	10–20 ul	15–20 min	Technically simple

RT-PCR—reverse transcriptase polymerase chain reaction; RT-qPCR—quantitative reverse transcriptase polymerase chain reaction; ELISA—enzyme-linked immunosorbent assay; IFA—immunofluorescent assay. IgG—immunoglobulin G; IgM—immunoglobulin M; IgA—immunoglobulin A. RBD—receptor-binding domain; N—nucleocapsid; E—envelope; S—spike; RdRp—RNA-dependent RNA polymerase proteins.

**Table 3:** Analytical assessment of antigen- and antibody-based diagnostic tests for SARS-CoV-2.

Study (Author, Ref)	Assay	Reference Comparator Assay	Sample Size	Results Dependent on Time from Symptom Onset/Age	Analyte Target	Sensitivity (95% CI)	Specificity (95% CI)
**Genotyping**							
Umunnakwe et al., 2022 [14]	Allplex^TM^ SARS-CoV-2 Variants II multiplex real-time PCR genotyping assay, Seegene (Seoul, South Korea)	Illumina MiSeq (Illumina Inc., San Diego, CA, USA), PacBio Sequel IIe (Pacific Biosciences Inc., Menlo Park, CA, USA) or Genexus Ion Torrent (Thermo Scientific, Waltham, MA, USA) platforms.	187	Not reported	K417N (Beta), K417T (Gamma). L452R (Delta), W152C (Epsilon)	Not reported	No cross reactivity
**RT-PCR**							
Marais et al., 2020 [15]	Rapid sample preparation for RT-PCR	Standard nucleic acid purification protocol for RT-PCR	195	Not reported	E, N and RdRp genes	41.7%–100% dependent on dilution factor PPA: 97.37% (92.55–99.28) NPA: 97.30% (90.67–99.52)	Not reported
Marais et al., 2022 [16]	RT-PCR saliva and mid-turbinate swab	Allplex ^TM^ 2019-nCoV SARS-CoV-2 PCR Abbott RealTime SARS-CoV-2 or Abbott Alinity m SARS-CoV-2 (Abbott Laboratories, Chicago, IL, USA)	453 [304 (Delta), 149 Omi-cron]	Yes	E, N and RdRp genes	Delta PPA on saliva: 73% (53–84) Omicron PPA on saliva: 96% PPA on mid-turbinate: 93%	Not reported
	FlowFlex SARS-CoV-2 N protein lateral flow assay	Allplex ^TM^ 2019-nCoV SARS-CoV-2 PCR Abbott RealTime SARS-CoV-2 or Abbott Alinity m SARS-CoV-2 (Abbott Laboratories, Chicago, IL, USA)	372 including 30 Delta, 29 Omi-cron	Yes	N gene	Delta variant: 93% Omicron variant: 68%	Not reported
Omar et al., 2021 [17]	Thermocycler (Genechecker; and 2400 SARS-CoV-2 Smartchecker PCR kit	Standard RT-PCR	315	Not applicable	N and RdRp genes	95% PPA: 82.4% NPA: 99.2%	97%
**Rapid antigen testing**							
Akingba et al., 2021 [18]	Abbott PanBio COVID-19 antigen RTD	Allplex ^TM^ 2019-nCoV SARS-CoV-2 PCR	677	Ct-dependent	N gene	69.17% (61.44–75.80)	99.02% (98.78–99.26)
Majam et al., 2022 [19]	Abbott PanBio COVID-19 antigen RTD	QuantStudio 5 Real-Time PCR System, Firmware version 1.3.3) using the TaqPath SARS-CoV-2 (Thermo Fisher Scientific, Waltham, MA, USA)	569	Ct-dependent	N gene	40% (30.3–50.3) PPA: 85.1% (71.7–93.8) NPA: 88.5% (85.5–91.1)	98.5% (96.9–99.4)
**Serological testing**							
Makatsa et al., 2021 [20]	Indirect in-house ELISA using recombinant plant-derived viral proteins	Euroimmun IgG S1	77	Not reported	Spike protein (S1 and RBD regions)	Reactivity same for both assays PPA: 89.4% (82.18–94.39) NPA: 88.4% (80.53–93.83)	Not reported
Gededzha et al., 2021 [21]	EUROIMMUN Anti-SARS-CoV-2 IgA and IgG	RT-qPCR	355	Not reported	Spike protein	IgG: 64.1% (59.1–69.0) IgA: 74.3% (69.6–78.6)	IgG: 95.2% (90.8–98.4) IgA: 84.2% (77–89.2)
Jugwanth et al., 2022 [22]	Abbott SARS-CoV-2 IgG Architect	RT-qPCR	526	Not reported	Nucleocapsid N protein	69.5% (64.7–74.1)	95% (89.9–98)
	Abbott SARS-CoV-2 Alinity	RT-qPCR	425	Not reported	Nucleocapsid N protein	64.8% (59.4–69.9)	90.3% (82.9–95.2)
	Abbott SARS-CoV-2 IgG Architect	In-house ELISA	197	Not reported	Spike protein	94.7% (88.8–98)	88.1% (79.2–94.1)
	Abbott SARS-CoV-2 Alinity	In-house ELISA	191	Not reported	Spike protein	92.5% (85.8–96.7)	91.7% (83.6–96.6)
Matefo et al., 2022 [23]	Laboratory-developed ELISA and IFA assay	Elecsys^TM^ Anti-SARS-CoV-2 ELISA and COVID-19 IgG/IgM Orient Gene	48	Not reported	Spike protein	ELISA: 100% IFA: 98.8% PPA for ELISA: 92.1% NPA for IFA: 91.0%	ELISA: 96% IFA: 100%
Grove et al., 2021 [24]	Roche Elecsys^TM^ chemiluminescent immunoassay	RT-PCR	434	Sensitivity increased >14 days	Nucleocapsid N protein	65.2% (59.57–70.46)	100% (97.07–100)
David et al., 2021 [25]	Zheihang OrientGene COVID-19 IgG/IgM	IgG versus PCR and IgG versus formal serology	150	Not reported	Spike protein	IgG versus PCR: 90.7% (81.7–96.2) IgG versus Formal Serology: 100% (94.5–100)	IgG versus PCR: 100% (95.2–100) IgG versus Formal Serology: 96.5% (90.0–99.3)
	Genrui Novel Coronavirus (2019-nCoV) IgG/IgM	IgG versus PCR and IgG versus formal serology	150	Not reported	Not reported	IgG versus PCR: 89.3% (80.1–95.3) IgG vs. Formal Serology: 98.5% (91.7–100)	IgG versus PCR: 97.3% (90.7–99.7) IgG versus Formal Serology: 94.1% (86.8–98.1)
	Boson Biotech 2019-nCoV IgG/IgM	IgG versus PCR and IgG versus formal serology	150	Not reported	Not reported	IgG versus PCR: 85.3% (75.3–92.4) IgG versus formal serology: 98.5% (91.7–100)	IgG versus PCR: 97.3% (90.7–99.7) IgG versus formal serology: 97.6% (91.8–99.7)
	Biosynex COVID-19 BSS	IgG versus PCR and IgG versus formal serology	150	Not reported	Not reported	IgG versus PCR: 84.3% (73.6–91.9) IgG versus formal serology: 98.2% (90.4–100)	IgG versus PCR:100% (91.4–100) IgG versus formal serology: 92.7% (82.4–98.0)
		In-house ELISA	111	Not reported	Spike protein	IgG: 80% (71.5–86.9) IgA: 87.8% (80.4–93.2)	IgG: 86.9% (77.8–93.3) IgA: 73.8% (63.1–82.8)
Shaw et al., 2021 [26]	Abbott SARS-CoV-2 IgG assay	Not reported	137	Not reported	Nucleocapsid N protein	Not reported	98.54% (94.82 -99.82)
Wolter et al., 2022 [27]	Wantai SARS-CoV-2 Ab ELISA	Elecsys^TM^ Anti-SARS-CoV-2 ELISA	7479	Not reported	Spike protein (RBD region)	91.0%	97.2%
Maritz et al., 2021 [28]	Semi-quantitative detection for IgG, IgM, IgA and Nab in serum	Not applicable	Not reported	Not reported	Spike protein (S1 region)	Range 83.2%–99.7%	Range 90.5%–99.1%
Kwatra et al., 2022 [29]	Dried blood spot sample serology vs. plasma serology	Not applicable	16	Not applicable	Spike protein (RBD region)	Correlation: RBD: 93.5% (81.4–97.8) S-protein: 96.5% (89.5–98.8)	Not reported
Irwin et al., 2021 [30]	2019-nCoV-IgG/IgM Rapid Test	Not reported	102	Age-dependent	Nucleocapsid N protein	IgM 67% IgG 69%	Not reported
	2019-nCoV IgG/IgM Rapid Test Cassette (whole blood, serum, or plasma),	Not reported	102	Age-dependent	Nucleocapsid N protein	IgM 15% IgG 65%	Not reported
	2019-nCoV Ab Test (Colloidal Gold)	Not reported	102	Age-dependent	Nucleocapsid N protein	IgM 13% IgG 36%	Not reported
	Medical Diagnostech COVID-19 IgG/IgM Rapid Test	Not reported	102	Age-dependent	Nucleocapsid N protein	IgM 26% IgG 66%	Not reported
	Cellex qSARS-CoV-2 IgG/IgM Cassette Rapid Test	Not reported	102	Age-dependent	Nucleocapsid N protein	IgM 64% IgG 67%	Not reported

95% CI—95% confidence interval; Ct—cycle threshold; PPA—positive per cent agreement; NPA—negative per cent agreement. RT-PCR—reverse transcriptase polymerase chain reaction; RT-qPCR—quantitative Reverse transcriptase polymerase chain reaction; ELISA—enzyme-linked immunosorbent assay; IFA—immunofluorescent assay. IgG—immunoglobulin G; IgM—immunoglobulin M; IgA—immunoglobulin A. RBD—receptor-binding domain; N—nucleocapsid; E—envelope; S—spike; RdRp—RNA-dependent RNA polymerase proteins.

## Data Availability

All relevant data are within the paper and could additionally be accessed through the relevant references included in the scoping review.
